# Curiosities

**Published:** 1860-02

**Authors:** George S. Fouke

**Affiliations:** Westminster, Md.


### CURIOSITIES.

MESSRS. TAFT & WATT.—*Gentlemen:* We have just had presented to us the rare phenomenon of *reproduced* enamel. We would report it for the consideration of *our* dental *savans*, who will no doubt be able to tell us whether the new-formed substance, in this "instance," be *bona fide* enamel? *osteo-dentine*? or a mere "*dental anomaly?*"

Mr. H., now residing in this county, while in the South some years ago, had an attack of yellow fever. "Snake root tea with *nitric acid* on it," was administered, but without using any protective for the teeth. The gentleman came out of the ordeal with his teeth "black as his hat." The upper central incisor was injured very much, a groove being cut across the labial surface, midway between the edge and neck of the tooth. Mr. H., expected nothing else but he should lose the tooth, and consequently sought no professional aid. To his great surprise, however, he observed his tooth began to fill up in the "deep groove." The process of *re-generation* continued till the incisor regained its original form.

Mr. H., possesses a "magnificent" set of masticators—a fact which has contributed, no doubt, to produce this anomalous occurrence. The *new formation* has every appearance of pure enamel!

In regard to the *possibility* of the enamel of a tooth being "*twice generated*," we are aware that such men as "George Waite, Esq.," have said such a thing can not be, for the simple reason, (according to their *theory*,) that "no parts are left to concur in the action" of *re-generation*. But, here is "a natural fact," and all we wish to have considered is, does it amount to "a demonstration?" May it not be that there are more "truths" in dental science, than are "dreamed of" in all *our* "philosophy?" *Magitot* says "the enamel, after the tooth is developed, undergoes a *very slow* organic movement," and he also says, that this tissue is subject to "changes of density and coloration." Under certain circumstances, (*propitious* for instance) might there not be a very "*rapid*" organic movement, which could produce the seemingly impossible "action" of re-produced enamel substance? Messrs. Editors, the stubborn "fact" before us, stumps us! Can't you help us out?

Another *curiosity* in dental "experience," met with lately, was a singular tooth we extracted for a young *negress*. It would puzzle a "Philadelphia" *dentist* to tell whether it be a monster *supernumerary* or two temporary incisors in a state of amalgamation. The tooth occupied a place behind the *permanent* incisors, and is a rare "conglomeration" of two teeth! Two incisor *crowns* are distinctly developed, and on the cutting edges form the letter *x*.

Our third and last *curious* case, is the existence of *Deciduous Teeth* at the age of twenty-six years. A gentleman in Taneytown, Md., flourishes *eight* lusty incisors in his upper maxilla. The teeth are in two regular rows, the *temporaries* the posterior ones, which are so firm and large as to be scarcely distinguishable from the permanent ones. When the jaws are closed, the lower incisors fit snugly between the two rows above, giving the possessor no inconvenience, and making his "bite" rather formidable, to be sure!

Sorry we have nothing just now more *practical* for your progressive pages, but if you esteem the above worthy of notice, you *might* confer an honor upon us by publishing our budget of "curiosities."

GEORGE S. FOUKE.

*Westminster, Md.*, Jan. 18th, 1860.

The stubborn fact should be very carefully examined. Consolidated dentine polished by prolonged friction or otherwise, sometimes assumes almost the appearance of enamel and might deceive on a slight examination. W.

